# Adaptive Filtering for Improved EEG-Based Mental Workload Assessment of Ambulant Users

**DOI:** 10.3389/fnins.2021.611962

**Published:** 2021-04-07

**Authors:** Olivier Rosanne, Isabela Albuquerque, Raymundo Cassani, Jean-François Gagnon, Sebastien Tremblay, Tiago H. Falk

**Affiliations:** ^1^Institut National de la Recherche Scientifique - Centre Énergie, Matériaux et Télécomunication, Université du Québec, Montréal, QC, Canada; ^2^Thales Research and Technology, Quebec City, QC, Canada; ^3^École de Psychologie, Université Laval, Quebec City, QC, Canada

**Keywords:** EEG, physical activity, amplitude modulation features, wearable sensors, adaptive filtering, mental workload assessment

## Abstract

Recently, due to the emergence of mobile electroencephalography (EEG) devices, assessment of mental workload in highly ecological settings has gained popularity. In such settings, however, motion and other common artifacts have been shown to severely hamper signal quality and to degrade mental workload assessment performance. Here, we show that classical EEG enhancement algorithms, conventionally developed to remove ocular and muscle artifacts, are not optimal in settings where participant movement (e.g., walking or running) is expected. As such, an adaptive filter is proposed that relies on an accelerometer-based referential signal. We show that when combined with classical algorithms, accurate mental workload assessment is achieved. To test the proposed algorithm, data from 48 participants was collected as they performed the Revised Multi-Attribute Task Battery-II (MATB-II) under a low and a high workload setting, either while walking/jogging on a treadmill, or using a stationary exercise bicycle. Accuracy as high as 95% could be achieved with a random forest based mental workload classifier with ambulant users. Moreover, an increase in gamma activity was found in the parietal cortex, suggesting a connection between sensorimotor integration, attention, and workload in ambulant users.

## 1. Introduction

Many professions, such as first responders (firemen, policemen, paramedics) and pilots are often faced with cognitive challenges including information overload, multitasking, interruptions, and fatigue. All these factors increase stress and reduce the efficiency with which this complex set of tasks is performed (Grtner et al., 2019). In many cases, these individuals are also exposed to a combination of physical and mental factors that further contribute to a high mental workload (MW), thus resulting in increased chances for errors, which could be life threatening. As such, MW monitoring has gained popularity in recent years.

Mental workload assessment can follow three methods: subjective, behavioral, or instrumental/objective. Subjective assessment relies on users reporting their perceived levels of mental workload and the NASA task load index (TLX) (Hart and Staveland, 1988; Cao et al., 2009) has been widely used. Behavioral methods, in turn, rely on task performance metrics (e.g., accuracy, response times, error rate) to characterize MW states. As can be seen, it is difficult for subjective and behavioral assessment methods to provide real-time measures of MW, thus have limited applications in closed-loop systems to improve task performance. This is where instrumental or objective methods have filled a gap. With such systems, real-time correlates of MW are obtained and unobtrusive neuronal and physiological measures have been explored, such as electroencephalography (EEG), electrocardiography (ECG), and galvanic skin response, amongst others.

With the popularization of wearable devices and improved dry electrode technologies, EEGs have emerged as a potential candidate for automated instrumental MW assessment (Lean and Shan, 2012; Mullen et al., 2015). Successful applications have been shown in aircraft pilots and car drivers (Borghini et al., 2014), and air traffic controllers (Aricò et al., 2016), to name a few. Numerous different features have been explored and shown useful, including power spectral, magnitude, and phase coherence (Aghajani and Omurtag, 2016; Dimitrakopoulos et al., 2017; So et al., 2017). For example, increases in theta and decreases in alpha band powers have been shown in prefrontal and parietal brain regions when task difficulty increases (Borghini et al., 2014). Temporal complexity measures have also shown some robustness against ocular and muscular artifacts (Tiwari et al., 2019) and spectro-temporal measures have been shown to provide complementary information to conventional power spectral ones (Albuquerque et al., 2019). Most available works, however, have relied on stationary users, such as sitting pilots and drivers (Borghini et al., 2014; Johnson et al., 2015), or have controlled for body movements (Hogervorst et al., 2014).

Practical applications, however, have users that are highly ambulatory (e.g., first responders). It is known that dry electrodes are very sensitive to movement artifacts, which could severely hamper MW monitoring performance (Morikawa et al., 2013). In our previous work, we explored the use of several conventional EEG enhancement algorithms to gauge their benefits in instrumental measurement of MW in highly ecological settings (Rosanne et al., 2019). We found that while some improvements were seen relative to using noisy raw data, overall MW measurement performance levels remained lower than what has typically been reported for stationary users. This is due to the fact that existing enhancement algorithms have been developed and optimized to remove muscle and eye blink/movement artifacts, and not necessarily movement artifacts seen with, e.g., running.

To overcome this limitation, here we propose the use of an adaptive filter to remove movement-specific motion artifacts from mobile EEG data. Accelerometry signals measured from the participant's torsos are used as reference signals for the adaptive filter. The algorithm was tested on a database collected in-house from 48 participants while they performed the Multi-Attribute Task Battery-II (Santiago-Espada et al., 2011) under two workload conditions (low and high) and two physical activity (PA) types (stationary bike and treadmill), each at three activity levels (none, medium, and high). Experimental results show the proposed algorithm accurately removing body movement artifacts and resulting in MW monitoring performance as high as 97% and independent of activity type and level.

Lastly, with the enhanced signals available, we conducted an in-depth analysis of the top features selected for MW assessment, thus obtaining insights into the cognitive processes involved during the workload task under physical activity. We found typical patterns related to visuo-motor control, attention, and fronto-parietal communication; patterns that would otherwise have been lost due to movement artifacts.

The remainder of this paper is organized as follows: section II describes the materials and methods used in the experiment. Section III presents and discusses the obtained results, and section IV presents the study conclusions.

## 2. Materials and Methods

### 2.1. Data Collection

Data was collected from 48 participants (23 females, 27.4 ± 6.6 year old), of which 22 utilized a treadmill during the experiment and 26 a stationary bike. Participants using the treadmill were asked to wear a safety harness around their chest in order to avoid falls. The experimental protocol was approved by the Ethics Boards at INRS and Université Laval, participants provided written consent, and were monetarily compensated for their time.

**Figure 1:**
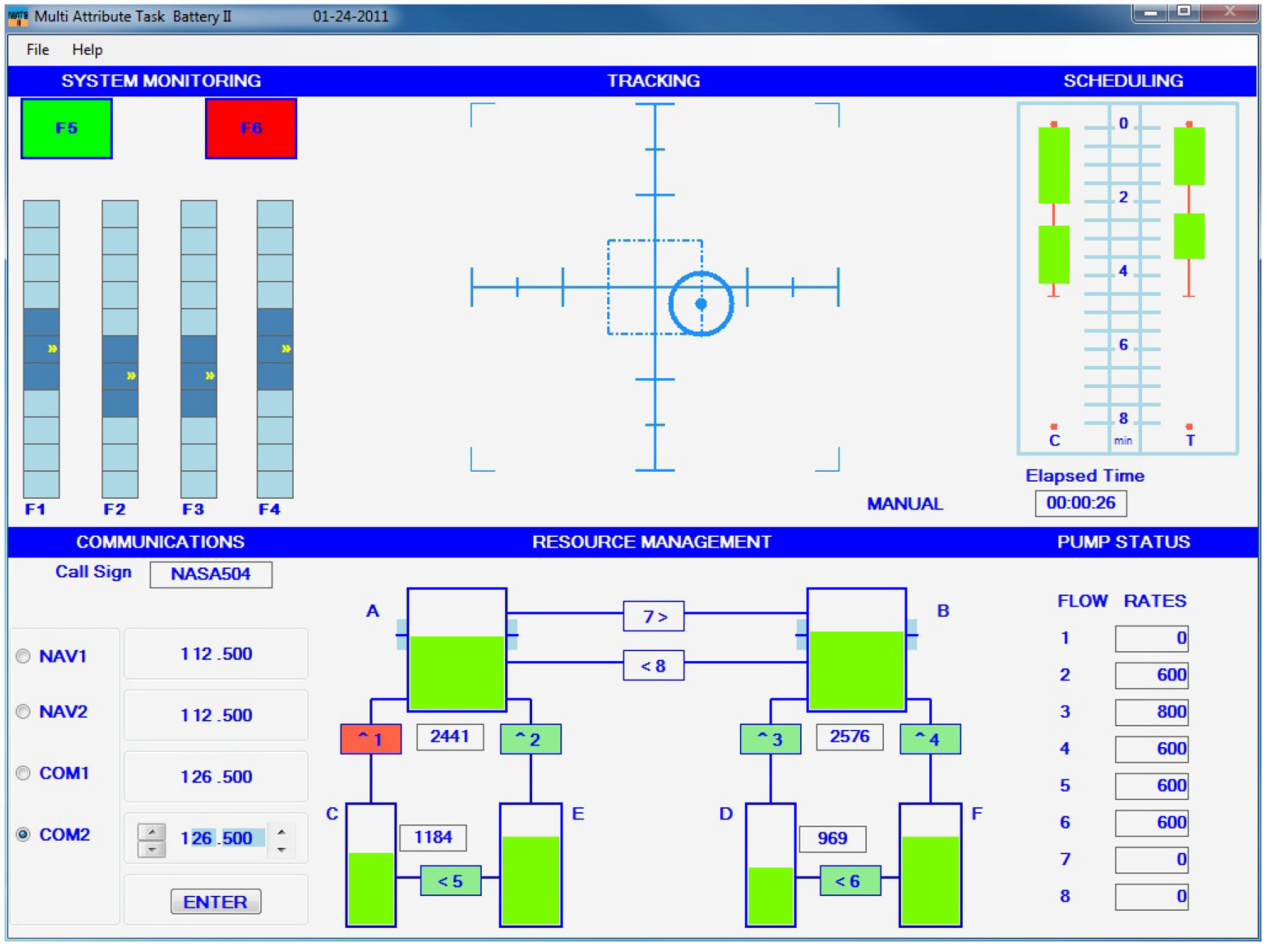
Graphical interface of the MATB-II software used to modulate high and low MW levels.

The experimental protocol comprised two MW levels (low/high) elicited through the MATB-II software, which has participants executing three simultaneous tasks: system monitoring, tracking, and resource management, as presented in Figure 1. Low and high MW settings were implemented based on changing the difficulty levels for each of the three tasks. As an example, a low MW task was composed by “easy” versions of the three tasks. Participants used an Xbox 360 joystick to interact with the MATB-II interface.

While executing MATB-II, subjects were asked to either bike or walk/jog on a treadmill at three levels of physical activity (PA): no movement, medium (treadmill: 3 km/h, bike: 50 rpm), and high (treadmill: 5 km/h, bike: 70 rpm). In total, six combinations of MW and physical activity were tested. The experiment was then split into six sessions, each one corresponding to one of the six combinations described above, counterbalanced to avoid ordering effects. Each session took 10 min to run and was systematically followed by a 5-min break. Before every session, two baseline periods were recorded. The first corresponded to 1 min without task nor physical activity. The second, in turn, corresponded to 1 min with only physical activity at the same level to be executed in the upcoming session. At the end of the experiment, each subject was asked to fill the NASA-TLX questionnaire (Hart and Staveland, 1988) to subjectively evaluate their perceived workload levels, as well as the reported their fatigue levels using the Borg scale (Borg, 1998).

**Figure 2:**
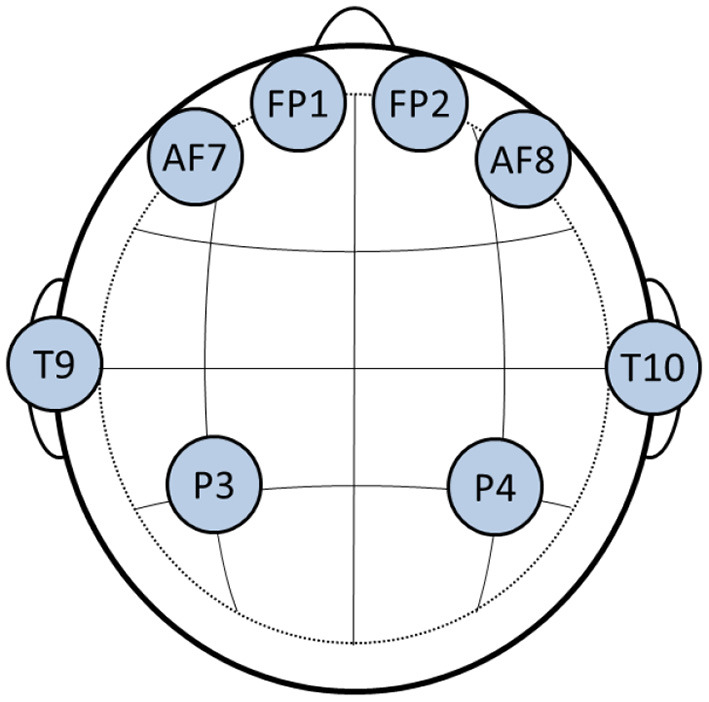
Electrode placement using the international 10–20 system.

EEG data was acquired from the participants using the Neurolectrics Enobio 8-channel portable headset with the following channel locations according to the international 10–20 system: Fp1, Fp2, AF7, AF8, T9, T10, P3, P4 (see Figure 2). Signals were collected at a sampling rate 500 Hz and were later downsampled to 250 Hz. Two virtual inter-hemispheric bipolar signals were also computed, namely Fp1-Fp2 and P3-P4. Movement activity was also recorded with a sampling rate of 50 Hz using the embedded accelerometer available in the Zephir Bioharness wearable device, which was placed on the chest of each subject. Accelerometry data was upsampled to 250 Hz to coincide with the EEG data. The interested reader is referred to Albuquerque et al. (2020) for more details about the database.

### 2.2. Movement Artifacts

**Figure 3:**
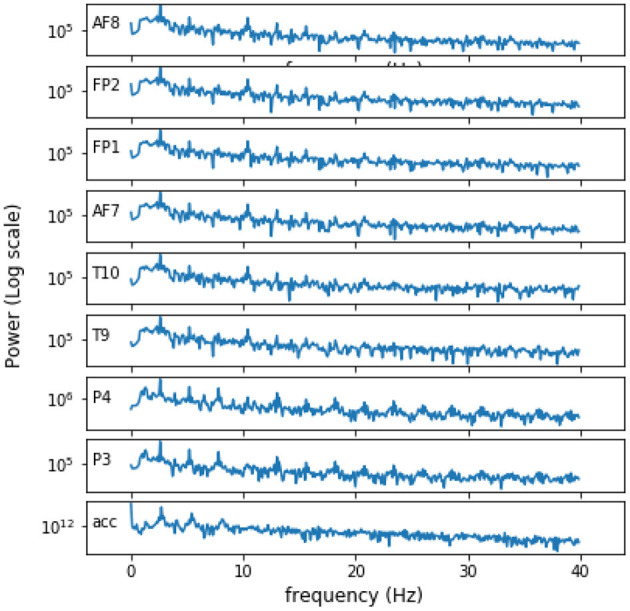
Average spectral representation of the eight EEG signals and the accelerometer signal over 10 s of recording for the low MW and high PA condition.

To illustrate the effects of movement, particularly in the walking/jogging conditions, Figure 3 depicts the average spectral representation of each of the eight EEG channels, as well as that of the accelerometer signals (bottom plot) during 10 s of the high physical activity condition. Here, the accelerometer signal corresponds to a L2-normalization of the accelerometer x, y, and z axes. As can be seen, particularly for the frequency range below 10 Hz, there is a significant effect from gait/movement on the EEG spectra, something previously reported in the literature (Zhang et al., 2014; Nathan and Contreras-Vidal, 2016). As movement artifacts are known to be detrimental to EEG quality (Gao et al., 2010; McMenamin et al., 2011), this has motivated the proposal of an adaptive filter using the accelerometer signal as a reference signal.

Movement artifacts observed in EEG signals can be caused either by a relative movement between the skin and the electrode (Burbank and Webster, 1978) or by a change in electrical potential when the skin stretches and contracts during movement (de Talhouet and Webster, 1996; Kearney et al., 2007). Movement artifacts have been reported to span spectral content between 0.11 and 20 Hz (Bouten et al., 1997), thus overlap with frequency bands relevant for mental workload monitoring (Mak et al., 2013). Conventional EEG enhancement algorithms, traditionally developed for ocular and muscle artifacts (Urigüen and Garcia-Zapirain, 2015; Mucarquer et al., 2019; Zou et al., 2019), have been shown to help with ambulatory users. For example, in Gwin et al. (2010), independent component analysis (ICA) and component-based template regression was used to remove gait movement artifacts from EEG event related potentials. ICA-based decomposition was also used to remove head movements in Onikura and Iramina (2015). Notwithstanding, these conventional solutions have been shown to interfere with MW assessment (Rosanne et al., 2019). Moreover, ICA-based enhancement methods typically rely on human intervention to remove artifactual components, thus have limited use in real-time applications. Adaptive filtering, in turn, has been used to reduce head movement artifacts (Mihajlović et al., 2014) and simulated random noise in EEGs (Raya and Sison, 2002). To the best of our knowledge, however, the use of adaptive filtering, with or without combined “blind” filtering approaches (i.e., that do not rely on human intervention), has yet to be quantified for EEG-based mental workload monitoring of ambulant users. We aim to fill this gap.

### 2.3. Adaptive Filtering

**Figure 4:**
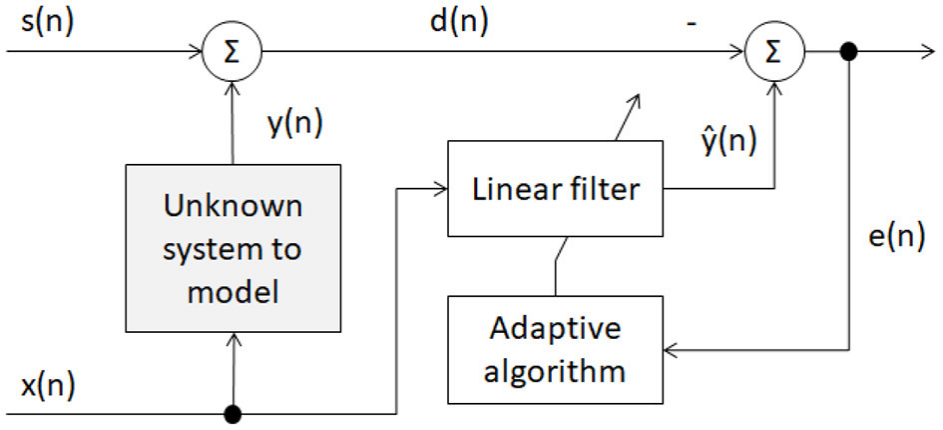
Block diagram of proposed adaptive filter.

Figure 4 depicts a block diagram of the adaptive filtering scheme explored herein. Signal *x*(*n*) corresponds to the accelerometer signal, whereas *s*(*n*) corresponds to the neuronal activity signal. From the accelerometer signal, movement artifacts are modeled and represent *y*(*n*). When added to the neuronal activity signal *s*(*n*), the output represents the noisy EEG signal *d*(*n*) = *s*(*n*) + *y*(*n*) recorded during physical activity. The goal of the adaptive filter is to find the optimal distortion weights $\hat{W}(n)$ from the accelerometer signal *x*(*n*) to best estimate the movement artifacts via $\hat{y}(n)$ and remove their effects from the noisy EEG signal via $e(n)=d(n)-\hat{y}(n)$.

More specifically:

(1) $$\begin{array}{l} \hat{y}\left(n\right)=\hat{W}\left(n\right)*x\left(n\right), \end{array}$$

and

(2) $$\begin{array}{l} e\left(n\right)=d\left(n\right)-\hat{y}\left(n\right),\\ e\left(n\right)=y\left(n\right)+s\left(n\right)-\hat{y}\left(n\right). \end{array}$$

The filter weights are found using the normalized least mean squares (NLMS) procedure (Diniz, 1997) for loss function *C*(*n*) using the steepest descent algorithm, i.e.:

(3) $$\begin{array}{l} \nabla_{{\hat{W}}^{H}}C\left(n\right)=\nabla_{{\hat{W}}^{H}}𝔼\left[e{\left(n\right)}^{2}\right]\\ \;=𝔼\left[2e\left(n\right)\nabla_{{\hat{W}}^{H}}e\left(n\right)\right]\\ \;=-2𝔼\left[x\left(n\right)e\left(n\right)\right], \end{array}$$

where ∇ is the gradient operator and 𝔼[·] the expected value. This leads to the following update rule:

(4) $$\begin{array}{l} \hat{W}\left(n+1\right)=\hat{W}\left(n\right)+\mu 𝔼\left[x\left(n\right)e\left(n\right)\right], \end{array}$$

where μ/2 is the step size.

We approximate the last term using the single-sample unbiased estimator $𝔼\left[x\left(n\right)e\left(n\right)\right]=\frac{x\left(n\right)e\left(n\right)}{|x\left(n\right){|}^{2}}$, thus simplifying (4) to:

(5) $$\begin{array}{l} \hat{W}\left(n+1\right)=\hat{W}\left(n\right)+\frac{\mu x\left(n\right)e\left(n\right)}{|x\left(n\right){|}^{2}}. \end{array}$$

**Figure 5:**
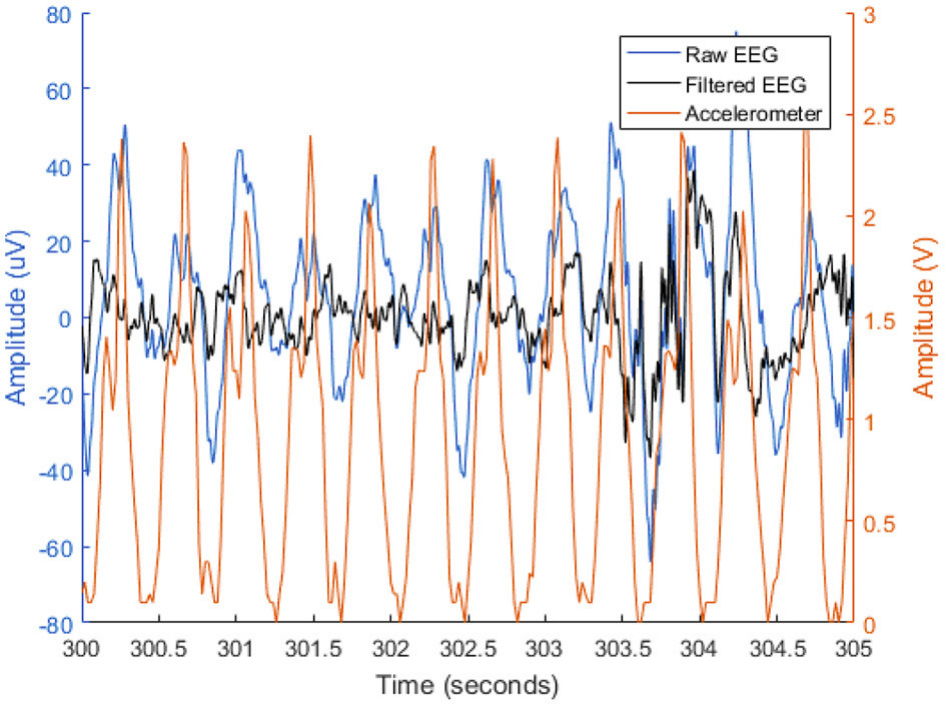
Time representation of a 5-s EEG segment from electrode AF8 before (blue) and after (black) adaptive filtering. The L2-normalization of the x, y, and z accelerometry axes is represented in orange.

Here, a filter length of 500 samples was used, corresponding to a signal duration of 2 s. Figure 5 depicts the noisy and enhanced EEG signals, as well as the accelerometry signal, to visually showcase the movement effects on the EEG signal and the effectiveness of the adaptive filter.

### 2.4. Benchmark Enhancement Algorithms

As mentioned previously, numerous EEG enhancement algorithms exist. Most have been developed to remove eye and muscular artifacts. Some are completely autonomous, whereas others rely on expert supervision. Here, four algorithms widely used for automatic (i.e., not relying on human intervention) EEG enhancement are used as benchmarks. They are used alone or in combination with each other. The following configurations are applied to the entire signals prior to epoching:

- **ASR:** artifact subspace reconstruction (ASR) is a method developed to remove transient and large-amplitude artifacts from noisy EEG. It relies on principal components analysis to reject large-variance components prior to reconstruction. The method relies on automatically identifying clean portions of the EEG signal and using these segments to determine thresholds for rejecting components. As stated in Chang et al. (2019), ASR has become the standard benchmark for EEG enhancement. The interested reader is referred to Mullen et al. (2015) for more details on the ASR method.
- **ADJUST:** Automatic EEG artifact Detection based on the Joint Use of Spatial and Temporal features (ADJUST) is an automatic artifact removal method that relies on “templates” of the effects of stereotyped artifacts (e.g., due to eye movements, blinks, and heart beats) on independent components. Components related to stereotyped artifacts are then removed and the signal is reconstructed. It has been reported that non-stereotyped artifacts, such as those due to movement, are not accurately removed with ADJUST and multiple methods are needed. More details about ADJUST can be found in Mognon et al. (2011).
- **Wavelet-ICA:** Wavelet-enhanced independent component analysis (ICA) relies on wavelet coefficient thresholding of independent components to reject artifactual components. The method has been shown to outperform conventional ICA and to better preserve EEG spectral and phase coherence properties (Castellanos and Makarov, 2006), especially for low-density EEG configurations (Cassani et al., 2014).
- **HAPPE:** The Harvard Automated Processing Pipeline for Electroencephalography (HAPPE) is a pipeline suitable for low density EEG channels and limited data samples. It relies on wICA and multiple artifact rejection algorithm (MARA) to detect artifactual components for rejection. The interested reader is referred to Gabard-Durnam et al. (2018) for complete details on the HAPPE method.
- **Algorithm Combinations:** In addition to the combined methods approach in HAPPE, the following additional benchmark algorithmic combinations were also explored: ASR + wICA and ASR + ADJUST. Moreover, the proposed adaptive filter was also used in combination with the benchmark algorithms to explore their combined effectiveness. Henceforth, results represented as “Raw” assume no enhancement, “AF” when only the adaptive filter has been applied, and methods combined with AF will be preceded by the prefix “AF_.”

### 2.5. Feature Extraction

Prior to feature extraction, EEG signals were first filtered with a FIR band-pass filter in the range 1–45 Hz. The following feature sets were extracted from the raw and enhanced signals:

#### 2.5.1. Power Spectral Density

Power Spectral Density (PSD) features measure signal power across different subband frequencies. In this study, nine frequency bands were considered, namely: δ (1–4 Hz), θ (4–8 Hz), α (8–12 Hz), β (12–30 Hz), low γ (30–45 Hz), δ to β (1–30 Hz), θ to β (4–30 Hz), low α (8–10 Hz), and high α (10–12 Hz). The relative power of each of these bands was calculated by normalizing per-band values by the full-band power. A total of 90 PSD features were extracted. Numerous studies have reported the usefulness of such features for mental workload assessment (Liu et al., 2017; Craik et al., 2019; Zhang et al., 2019).

#### 2.5.2. Phase and Magnitude Spectral Coherence

Phase and Magnitude Spectral Coherence (PMSC) features are useful for measuring connectivity between cortical regions as these techniques measure co-variance of the phase and magnitude between two signals. The interested reader is referred to Aoki et al. (1999) for more details on PMSC computation. PMSC is computed for two pairs of electrodes, namely FP1-FP2 and P3-P4 for each 5 sub-bands (δ, θ, α, β, γ). A total of 20 PMSC features were extracted. These features are motivated from Zhang et al. (2014) and Zarjam et al. (2015) that have shown their usefulness in mental workload assessment.

#### 2.5.3. Amplitude Modulation Rate-of-Change

Amplitude Modulation (AM) rate-of-change features quantify the rate-of-change of specific frequency sub-bands and provides insight into cross-frequency magnitude-magnitude coupling/interactions and reveals interactions between different brain processes (Tort et al., 2010; Voytek et al., 2010; Seeber et al., 2014), as well as long-range communication (Zanto et al., 2011; Clayton et al., 2015). The interested reader is referred to Trambaiolli et al. (2011) and Fraga et al. (2013) for more complete details on the measure. A total of 140 features were extracted that provide robustness against movement artifacts, as described in Albuquerque et al. (2018).

#### 2.5.4. Phase and Magnitude Spectral Coherence of Amplitude Modulation Features (PMSC-AM)

PMSC-AM extends the capacity of PMSC features to amplitude modulations. These features were recently proposed for affective state monitoring and showed useful for arousal and valence prediction (Clerico et al., 2015, 2018). They are explored here for the first time as correlates of mental workload. These features are based on the modulated signals of each band which make a total of fourteen signals per channels (see Clerico et al., 2018 for more details). After splitting the signals into epochs, the magnitude spectral coherence and phase coherence is then computed for the FP1-FP2 and P3-P4 channel pairs only. A total of 56 features were extracted.

### 2.6. Feature Selection and Ranking

Feature selection is a common step in classification tasks to remove redundant (Peng et al., 2005) or irrelevant features (Blum and Langley, 1997) and for dimensionality reduction (Fan and Fan, 2008) to improve classification performance. In this study, we rely on the so-called minimum Redundancy Maximum Relevance (mRMR) filter method (Peng et al., 2005) which not only finds the most relevant features for the task at hand, but removes features with high mutual information, thus minimizing redundancy. The algorithm has been shown to be extremely useful for EEG-based affective state assessment (e.g., Cassani et al., 2014; Clerico et al., 2018). In addition to feature selection, we further rank the importance of the top-features using a wrapper-based method. It is important to emphasize that feature selection/ranking is not crucial here, given the number of features explored. Nonetheless, we use it to obtain insights into the neuronal patterns related to mental workload during activity and how such patterns may be affected by movement artifacts.

### 2.7. Classification and Hyperparameter Tuning

We are interested in exploring the effects of movement artifacts and, consequently, EEG enhancement on mental workload assessment. Here, we assume the binary problem of classifying low vs. high mental workload levels. Two conventional classifiers are explored, namely random forest (RF) (Qi, 2012) and support vector machine (SVM). A repeated (10 times) 10-fold cross validation testing setup is used.

For hyperparameter tuning, the cross-validation grid search available in the scikit-learn library (Pedregosa et al., 2011) was explored. This approach, however, yielded a high number of trees (around 500) for the RF classifier, as compared to the amount of available data (Oshiro et al., 2012). As an alternative, we empirically fixed tree depth to 8 and stopped adding trees once the evolution of the area under the curve—receiver operating characteristics (AUC-ROC) became constant across out-of-bag conditions. Next, a similar strategy was used to optimize tree depth and we fixed the number of trees to the value found in the previous analysis. In both cases, a stratified 5-fold cross-validation procedure was used with all subjects to ensure reliable generalization performance.

**Figure 6:**
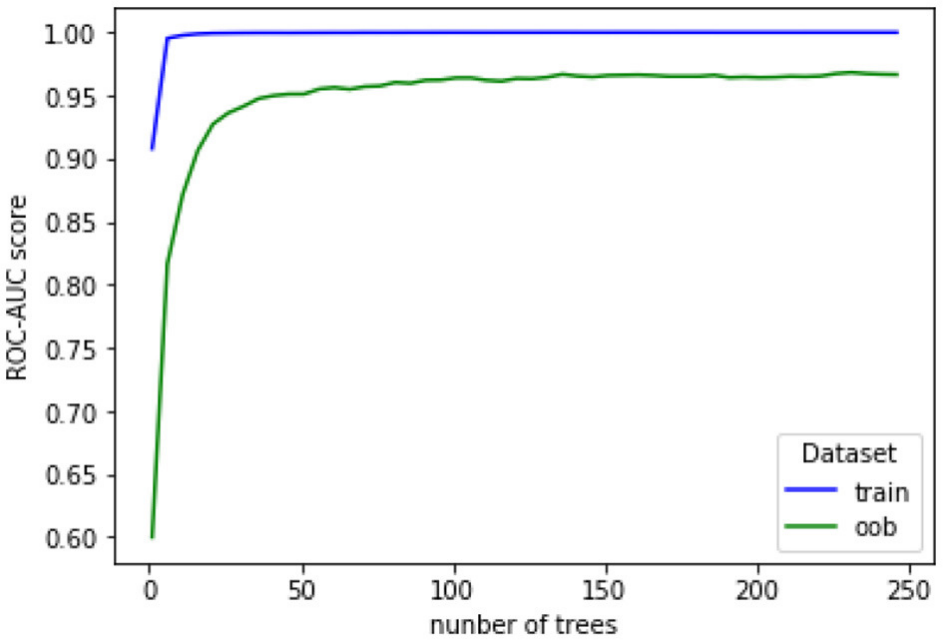
Evolution of AUC-ROC for training and out-of-bag (oob) sets as a function of number of trees.

**Figure 7:**
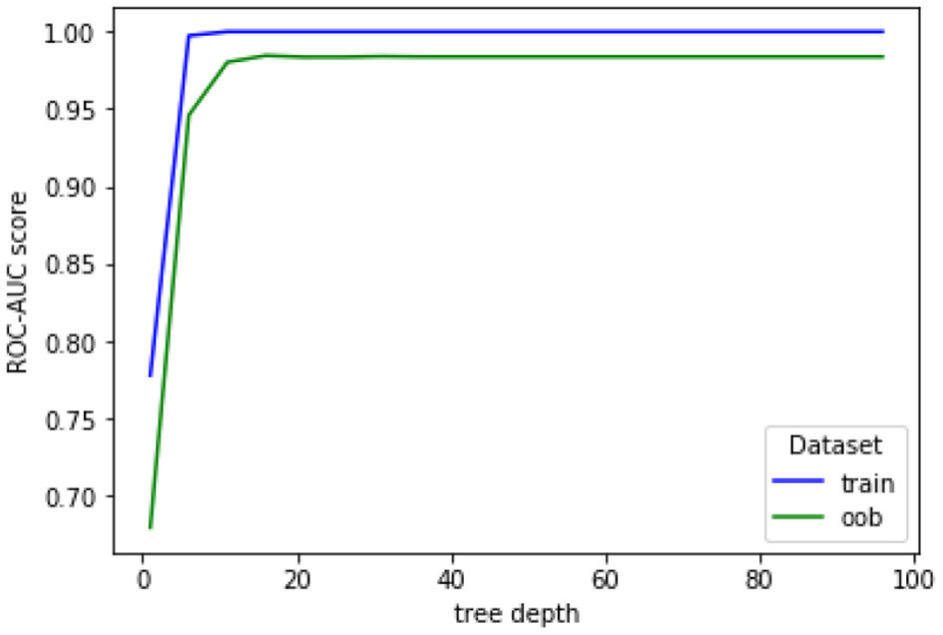
Evolution of AUC-ROC for training and out-of-bag (oob) sets as a function of tree depth.

Figure 6 shows the evolution of AUC-ROC scores for the training and out-of-bag (oob) sets as a function of number of trees. When building each random tree in the forest, not all features and samples of the dataset are used. Instead, a small randomly-selected set called the bootstrap bag is used to build a single tree; this bag is different for each tree. The oob set, thus, corresponds to the remaining unused samples. The accuracy with the oob set is shown to stabilize at around 100 trees. Moreover, Figure 7 depicts accuracy as a function of tree depth. As can be seen, for the out-of-bag set the accuracy plateaus at around a depth of 10. Henceforth, these values are used in our experiments.

**Figure 8:**
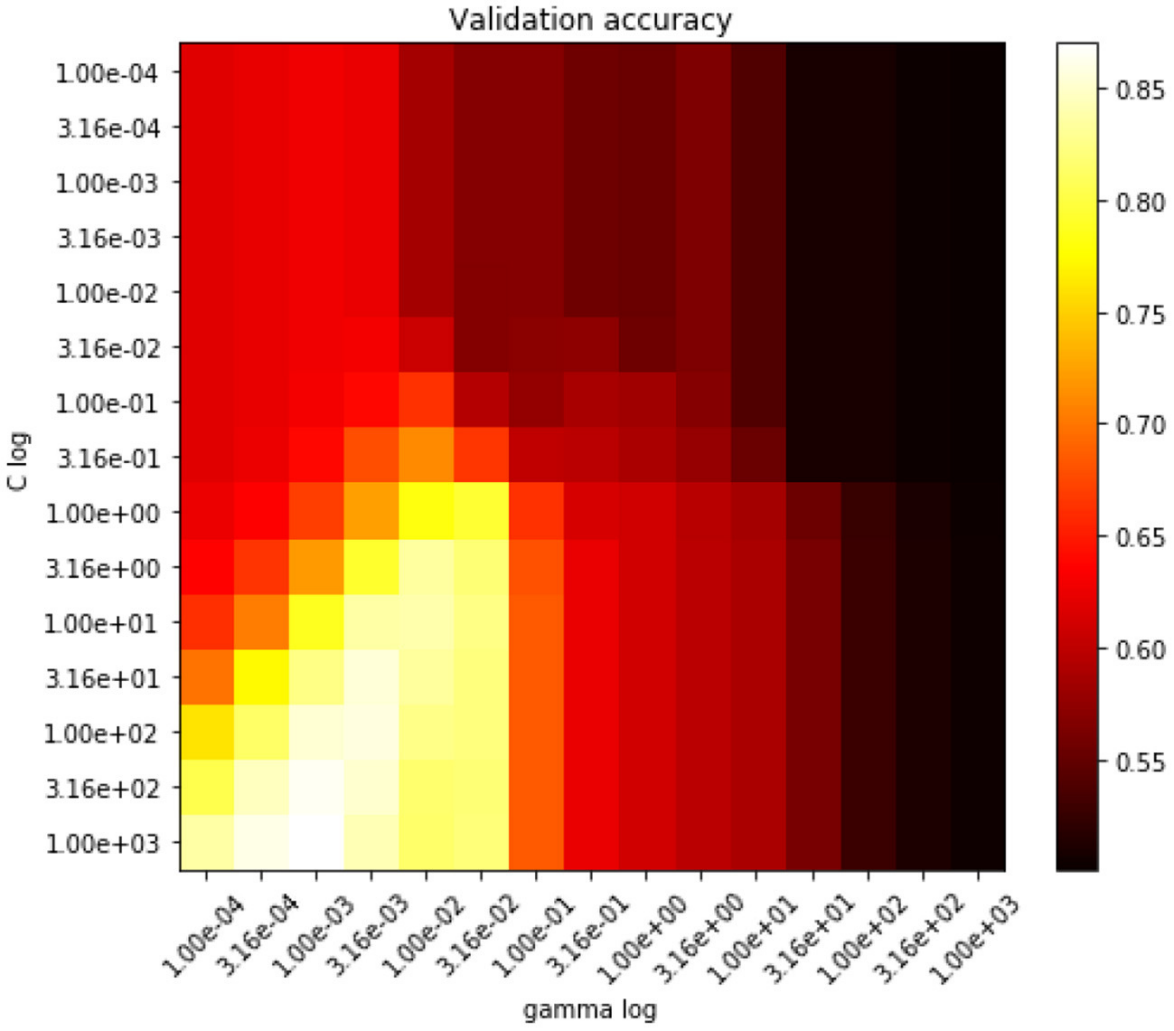
Accuracy hyperparameter grid search for the SVM classifier.

Figure 8 presents accuracy values obtained during a grid search to find the optimal *C* and γ values of the support vector classifier. It can be seen that the best accuracy is reached with *C* = 310 and γ = 0.001 with a Radial Basis Function kernel; these values are used henceforth.

## 3. Results and Discussion

### 3.1. Classification Performance

#### 3.1.1. Ablation Study

In order to estimate the impact of the adaptive filter on EEG enhancement, mental workload classification accuracy is reported with and without its use. Tables 1, 2 present classification accuracy values for the RF and SVM classifiers, respectively. While each column corresponds to a tested benchmark enhancement algorithm, with or without (termed “Base”) adaptive filtering, each row corresponds to a specific feature set used for classification in the low and high physical activity (PA) conditions. Row labeled “All” indicates fusion of all features. Results reported are the average of a 10-fold cross-validation test setup repeated ten times by shuffling the partitions each time. Whenever the achieved results with the adaptive filter were significantly different (based on a paired *t*-test) than without, results are indicated with superscripts “†” and “‡” for *p* ≤ 0.05 and *p* ≤ 0.01, respectively.

**Table 1:** RF mental workload classification accuracy for different feature and enhancement algorithm configurations.

**Random forest**		**Raw**		**ASR**		**ADJUST**		**HAPPE**		**ASR_ADJUST**		**ASR_wICA**		**wICA**	
		**Base**	**AF**	**Base**	**AF**	**Base**	**AF**	**Base**	**AF**	**Base**	**AF**	**Base**	**AF**	**Base**	**AF**
PSD	low PA	86.27	85.59^‡^	88.60	89.71^‡^	89.23	88.89	86.25	92.96^‡^	93.68	90.37^‡^	92.34	92.73	88.66	90.87^‡^
	high PA	87.15	85.05^‡^	89.55	89.89	89.92	89.76	89.57	90.91^‡^	89.14	91.80^‡^	94.13	94.61^‡^	89.14	91.69^‡^
AM	low PA	83.91	84.28^†^	84.78	86.48^‡^	88.12	88.26	83.90	91.93^‡^	91.94	87.28^‡^	87.26	89.47^‡^	84.83	87.88^‡^
	high PA	83.83	84.77^‡^	86.84	89.21^‡^	88.27	89.08^‡^	87.33	89.36^‡^	87.62	89.13^‡^	90.91	92.60^‡^	85.59	89.27^‡^
PMSC	low PA	84.23	85.76^‡^	82.67	82.66	90.05	92.26^‡^	87.85	95.15^‡^	89.38	87.08^‡^	84.33	82.44^‡^	84.66	86.03^‡^
	high PA	82.07	82.78^‡^	80.74	79.95^‡^	86.79	88.08^‡^	89.80	90.51^‡^	89.90	84.84^‡^	82.44	80.46^‡^	81.31	83.81^‡^
PMSC-AM	low PA	65.79	70.81^‡^	67.89	68.32	66.62	71.92^‡^	73.84	78.34^‡^	67.77	64.99^‡^	70.18	67.48^‡^	65.56	70.19^‡^
	high PA	67.90	74.10^‡^	67.57	67.66	69.59	70.56^‡^	71.46	75.71^‡^	68.46	64.78^‡^	67.75	67.05^†^	68.59	72.09^‡^
All	low PA	89.17	95.03^‡^	90.23	94.32^‡^	92.55	95.65^‡^	93.24	97.90^‡^	96.21	93.56^‡^	93.61	95.86^‡^	90.49	96.22^‡^
	high PA	88.89	91.20^‡^	90.77	93.39^‡^	94.22	95.36^‡^	94.54	97.89^‡^	93.36	93.54	94.95	95.19	90.20	93.97^‡^

**Table 2:** SVM mental workload classification accuracy for different feature and enhancement algorithm configurations.

**SVM**		**Raw**		**ASR**		**ADJUST**		**HAPPE**		**ASR_ADJUST**		**ASR_wICA**		**wICA**	
		**Base**	**AF**	**Base**	**AF**	**Base**	**AF**	**Base**	**AF**	**Base**	**AF**	**Base**	**AF**	**Base**	**AF**
PSD	low PA	59.31	59.35	61.72	60.16^‡^	64.08	67.16^‡^	67.98	73.57^‡^	70.72	71.37	66.66	63.13^‡^	59.99	59.82
	high PA	64.22	66.56^‡^	65.78	67.81^‡^	68.40	70.00^‡^	67.56	73.58^‡^	67.61	71.91^‡^	69.29	70.96^‡^	64.79	70.07^‡^
AM	low PA	56.75	59.03^‡^	58.37	61.05^‡^	61.63	69.18^‡^	62.69	73.39^‡^	69.48	68.65^†^	60.74	62.74^‡^	56.15	59.63^‡^
	high PA	62.49	65.35^‡^	66.67	64.64^‡^	68.88	69.66^‡^	66.16	70.94^‡^	68.45	70.36^‡^	68.80	66.77^‡^	63.27	67.22^‡^
PMSC	low PA	60.25	72.25^‡^	60.70	69.61^‡^	61.59	68.45^‡^	63.94	75.11^‡^	70.45	68.22^‡^	61.06	69.71^‡^	61.07	73.27^‡^
	high PA	60.43	71.86^‡^	65.79	68.48^‡^	67.97	69.62^‡^	70.80	72.98^‡^	72.09	67.64^‡^	66.35	69.04^‡^	60.05	71.34^‡^
PMSC-AM	low PA	56.05	62.53^‡^	56.57	58.89^‡^	56.03	62.04^‡^	59.83	65.23^‡^	59.37	58.68^†^	56.60	58.04^‡^	55.36	59.79^‡^
	high PA	59.35	65.37^‡^	59.63	60.29	61.36	61.69	59.11	63.43^‡^	58.49	58.61	59.96	62.62^‡^	60.10	64.21^‡^
All	low PA	64.94	78.34^‡^	66.59	73.54^‡^	73.28	80.25^‡^	78.88	87.49^‡^	76.60	79.58^‡^	68.22	75.37^‡^	66.87	77.39^‡^
	high PA	71.37	81.09^‡^	73.31	76.26^‡^	78.33	81.03^‡^	79.38	86.93^‡^	78.94	77.88^‡^	74.42	77.72^‡^	71.22	81.86^‡^

As can be seen from the Tables 1, 2, the adaptive filter significantly improved accuracy for most tested configurations, particularly for features derived from the amplitude modulation analysis, as well as for the high physical activity conditions in which movement artifacts are most pronounced. Overall, the RF classifier consistently outperformed the SVM.

For PSD based features, the best results were achieved with a combination of ASR and ADJUST methods (93.68%), followed closely by HAPPE and AF (92.96%) for low physical activity conditions and the ASR-wICA-AF combination for high PA conditions. Similar accuracy values were achieved for the AM and PMSC feature sets. The PMSC-AM features, on the other hand, resulted in the lowest values, thus suggesting that they may not be useful for mental workload assessment when used alone. Overall, fusion of the different feature sets showed to result in the highest accuracy for both RF and SVM classifiers, thus suggesting their complementarity. The highest accuracy achieved was of 97.90% with the HAPPE-AF combination for both the high and low PA conditions. Such findings show that by combining all feature sets with the proposed adaptive filtering and HAPPE enhancement methods, the same mental workload measurement accuracy can be achieved despite physical activity levels.

#### 3.1.2. Effect of Number of Features

**Figure 9:**
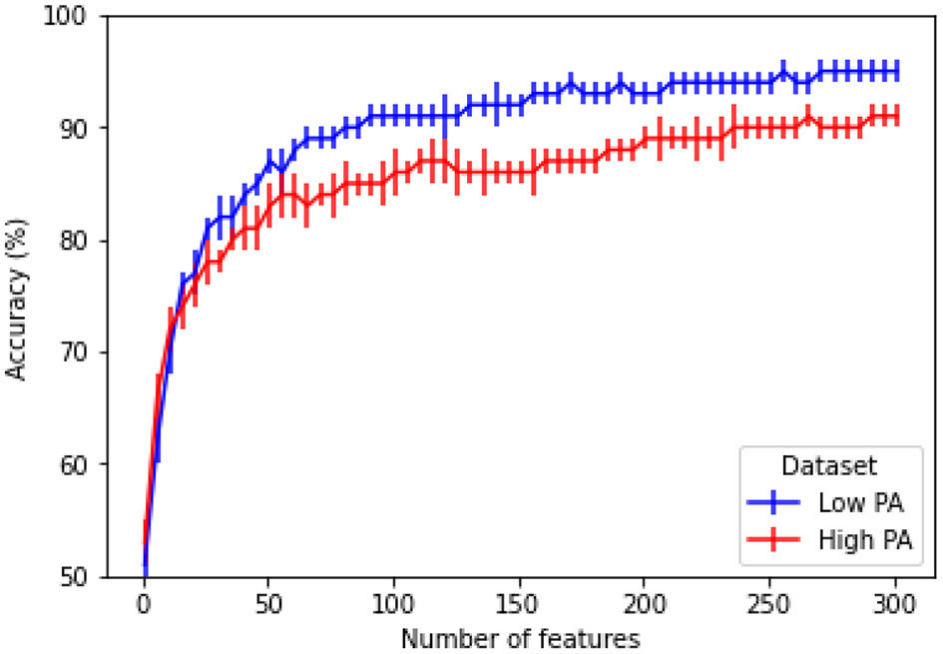
Accuracy vs. number of features for a RF classifier and a combined AF-HAPPE enhancement pipeline.

The results reported in Tables 1, 2 relied on all extracted features. In order to investigate the impact of feature dimensionality on overall accuracy, Figure 9 depicts the achieved accuracy as a function of number of features used, in decreasing importance, as ranked by mRMR. Here, the AF-HAPPE enhancement combination is used with the RF classifier and the average accuracy over a single 10-fold cross-validation setup is used. For this comparison, default classifier parameters are used in order to gauge the effectiveness of the features *per se*, and not the classifier. As can be seen, sharp increases in accuracy are achieved with the first 60 features and then slight increases occur after 100 and then 200 features are considered. A small gap is seen for both low and high physical activity conditions once all 306 features are used. If feature dimensionality is of concern, the achieved results and the small gap between low and high PA conditions suggest that 236 features can be a good compromise (94 and 90%, low and high PA, respectively), followed by 111 features (91 and 87%, low and high PA, respectively). For comparison, with the top-60 features, accuracy of 84 and 88% are achieved, respectively.

### 3.2. Top-Ranking Features

To obtain insights from top-selected features, we performed an in-depth analysis of the top-60 features selected from the combined “All” feature set in the low and high physical activity conditions using both the raw data and the top-performing AF_HAPPE enhanced data; Table 3 lists these features.

**Table 3:** Top-60 features for different physical activity (PA) and signal processing conditions.

**Raw**		**AF_HAPPE**	
**Low PA**	**High PA**	**Low PA**	**High PA**
β-mα-P4	msc-β-mθ-FP1-FP2	γ-mγ-FP1-FP2	phc-β-mδ-FP1-FP2
α-mδ-AF8	msc-δ-P3-P4	δ-P3-P4	γ-mδ-P4
tab-FP1	γ-mδ-T10	γ-mβ-P4	β-mδ-T9
α-AF8	α-mθ-T10	α1-T9	γ-mθ-AF8
θ-mθ-FP1	α-mδ-FP1	β-mα-AF7	β-mθ-AF8
γ-mδ-P4	γ-mθ-FP2	θ-mθ-T9	β-mθ-P4
γ-mθ-T10	β-mδ-AF7	γ-mδ-P4	γ-mδ-AF8
θ-mθ-T9	β-mθ-P4	dtab-T10	θ-mθ-P3-P4
θ-mθ-FP1-FP2	β-mδ-FP1-FP2	β-mθ-AF7	tab-FP2
θ-mθ-T10	β-mδ-T10	θ-P3-P4	γ-mα-FP1-FP2
α1-FP1-FP2	α-mθ-AF8	δ-mδ-FP1	α-T9
α-mθ-P3	α-mδ-P3	γ-mθ-FP2	γ-mδ-FP2
γ-mθ-FP2	msc-α-mδ-FP1-FP2	γ-mδ-P3	β-T9
γ-mβ-FP2	α-FP2	tab-T9	msc-γ-mδ-FP1-FP2
θ-mδ-FP1-FP	β-mα-P4	β-mδ-P3	β-mδ-FP2
α-mθ-P3-P4	α-AF8	γ-mδ-FP2	α2-P4
γ-mα-P3	θ-mδ-FP1-FP2	dtab-T9	dtab-P3
dtab-T9	α2-T10	θ-mθ-FP1-FP2	γ-mθ-T10
β-mβ-P3-P4	θ-FP2	γ-mβ-AF8	β-mθ-FP2
tab-T9	tab-FP2	γ-mδ-FP1	γ-P3
δ-mδ-AF7	α2-T9	β-P3	β-mθ-T9
phc-δ-mδ-P3-P4	β-mθ-P3	γ-mγ-FP2	γ-mθ-FP2
γ-mβ-P4	γ-mα-P3-P4	θ-mδ-FP1	γ-mβ-P3
β-mβ-T9	γ-mα-FP2	phc-β-mθ-P3-P4	γ-FP1
β-mβ-P3	θ-T10	γ-mγ-P3	δ-AF8
dtab-AF7	β-mδ-P3-P4	θ-mθ-P3	β-mα-FP1-FP2
γ-mα-FP2	dtab-T9	γ-mγ-FP1	γ-mθ-FP1-FP2
θ-T10	β-T10	β-mθ-FP1	γ-mθ-P4
γ-mα-T9	tab-T10	γ-mγ-P3-P4	δ-mδ-FP1
θ-mθ-AF8	γ-T9	γ-mβ-FP1-FP2	β-mα-AF7
γ-mθ-P3	θ-mθ-P3	β-mα-P3	θ-mδ-P4
γ-T10	dtab-FP1	γ-mθ-FP1	msc-β-mθ-FP1-FP2
δ-AF7	β-P3	α2-T9	δ-mδ-AF8
δ-mδ-P3-P4	msc-γ-FP1-FP2	β-mδ-P4	tab-FP1-FP2
α-mθ-T10	msc-α-mθ-FP1-FP2	δ-mδ-P4	β-AF8
β-mδ-P3-P4	α-mθ-FP2	δ-P4	β-mθ-P3
γ-mθ-P3-P4	α1-P3-P4	phc-θ-P3-P4	β-P3-P4
β-mδ-P3	γ-mδ-P3-P4	γ-mβ-FP1	γ-mδ-T9
α-T10	δ-mδ-P4	γ-mα-FP1-FP2	α1-T10
α2-P3-P4	γ-P3	θ-mδ-P3	δ-mδ-FP2
θ-mδ-P4	α1-T9	β-mα-P4	msc-β-mβ-FP1-FP2
β-mθ-P3-P4	β-mθ-P3-P4	β-mθ-P3-P4	β-mδ-P4
θ-mδ-T10	α-mδ-FP2	β-mβ-P4	α-T10
msc-β-mδ-P3-P4	γ-T10	γ-mθ-FP1-FP2	γ-mγ-T9
phc-β-P3-P4	β-P3-P4	β-mβ-FP2	msc-δ-P3-P4
α-mδ-P4	msc-β-mδ-FP1-FP2	γ-mα-FP1	msc-β-mα-FP1-FP2
γ-FP1-FP2	δ-FP1-FP2	δ-mδ-P3-P4	msc-γ-mθ-FP1-FP2
θ-P3-P4	msc-γ-mθ-FP1-FP2	γ-mβ-FP2	γ-mα-P3-P4
phc-δ-P3-P4	β-P4	δ-FP1-FP2	γ-mα-T9
α1-P3-P4	tab-P4	γ-mβ-P3-P4	δ-mδ-P4
β-mδ-T9	δ-T9	β-mβ-FP1-FP2	msc-δ-FP1-FP2
α1-T9	dtab-T10	γ-T10	β-mδ-AF7
γ-mδ-P3-P4	α-mδ-T10	γ-mγ-T10	β-mβ-P3-P4
θ-P4	msc-β-FP1-FP2	γ-mβ-T10	γ-mθ-AF7
α2-P4	msc-θ-FP1-FP2	γ-mα-P3-P4	γ-mθ-P3-P4
dtab-P3	dtab-P4	β-mδ-P3-P4	γ-mβ-P3-P4
θ-FP2	δ-P3-P4	θ-mδ-P3-P4	msc-θ-FP1-FP2
β-FP2	tab-T9	α-mδ-T9	β-mθ-AF7
α2-T9	msc-δ-FP1-FP2	dtab-FP1	msc-α-FP1-FP2
α-P3	β-FP2	γ-mα-T10	phc-γ-FP1-FP2

*Feature names are self explanatory and follow the feature-electrode notation; “tab” corresponds to 4–30 Hz spectral subband power; “dtab” to 1–30 Hz; “phc” to phase coherence; and “msc” to magnitude square coherence*.

As can be seen, for all conditions tested, modulation spectral features resulted in the majority of the top 60 features. For example, for the high PA conditions without and with AF_HAPPE processing, they corresponded to 50 and 70% of the top features, respectively. This corroborates findings from Albuquerque et al. (2018, 2019) and Clerico et al. (2018) that show the importance of such features for mental workload and affective state assessment, as well as their robustness to movement artifacts.

Coherence based measures, in turn, were the second top-performing features and appeared mostly in high PA conditions. They represented ~17 and 18% of the top features for the raw and enhanced conditions, respectively. Coherence measures have been linked movement and visual-motion discrimination and are indicative of the additional mental resources involved during physical activity (Händel and Haarmeier, 2009; Cheron et al., 2016). The important coherence features were mostly extracted from the pre-frontal regions, which have been linked to mental workload and attention (Mandrick et al., 2013), while a few were extracted from parietal regions, thus suggesting some contribution of balance control also involved (Hülsdünker et al., 2015).

Regarding brain hemispheres, features from the right regions were selected slightly more often than the left hemisphere, particularly in high PA conditions. This corroborates previous work (Perennou et al., 1999) that has shown the existence of a right hemispheric dominance for postural control. Inter-hemispheric signals, in turn, corresponded to roughly 33% of the top features for all PA conditions. Within the top features, inter-hemispheric parietal features typically appeared in low PA conditions, whereas inter-hemispheric pre-frontal features appeared during high PA conditions. This suggests a shift in visuo-motor (Iacoboni and Zaidel, 2004) and attention (Vossel et al., 2016) aspects during low PA, to more complex motor behaviors and sensorimotor integration aspects with high PA (Geschwind and Iacoboni, 1999). Overall, in the enhancement scenario, the parietal regions were responsible for the majority of the top features, followed closely by the pre-frontal cortex, for both low and high PA conditions. These results are in line with the classical mental workload literature with non-ambulant users (Aoki et al., 1999; Holm et al., 2009; Borghini et al., 2012; Mandrick et al., 2013; Käthner et al., 2014; Al-Shargie, 2019), thus further showing the promise of the proposed adaptive filtering scheme.

Lastly, regarding EEG subband frequencies, as expected, adaptive filtering combined with HAPPE reduced the importance of features extracted from θ and α bands, as these have the highest overlap with the accelerometry data. It did, on the other hand, boost the importance of features extracted from the β and γ bands. It is well-known that γ is highly sensitive to muscle activity (Muthukumaraswamy, 2013) and HAPPE is known to remove such artifacts. With the proposed enhancement scheme, γ features (and γ−*mδ*) remained consistent between low and high PA conditions and covered aspects related to sensory motor integration (Aoki et al., 1999; Sauseng et al., 2015), attention (Sammer et al., 2007; Wang et al., 2017), and balance control (Gwin et al., 2011; Sipp et al., 2013). The importance of the β band, in turn, has been observed in other studies during intense physical exercises (Rahman et al., 2019), anticipation in a decision making game (Cohen et al., 2009) and increment of cognitive control and attention (Kakkos et al., 2019).

## 4. Conclusions

This paper has proposed the use of an adaptive filtering scheme to remove movement artifacts from EEG signals for robust mental workload assessment. Experimental results have shown that the proposed adaptive filtering scheme is best combined with HAPPE and can result in 97% mental workload prediction accuracy for both low and high physical activity conditions. Moreover, an in-depth analysis of the top-selected features have shown the importance of modulation spectral features for the task at hand, as well as the potential of the proposed enhancement solution at maintaining important discriminant information from the EEG for mental workload measurement, in particular those captured by γ frequency band-based features.

## Data Availability Statement

The raw data supporting the conclusions of this article will be made available by the authors, without undue reservation.

## Ethics Statement

The studies involving human participants were reviewed and approved by Ethics Review Boards of INRS, Universite Laval, and the PERFORM Centre (Concordia University). The patients/participants provided their written informed consent to participate in this study.

## Author Contributions

OR, IA, RC, and J-FG: statistical analysis and programming. OR, IA, and RC: data collection. ST and TF: funding and supervision. All authors experimental design, writing, and reviewing.

## Conflict of Interest

J-FG was employed by the company Thales Research and Technology. The remaining authors declare that the research was conducted in the absence of any commercial or financial relationships that could be construed as a potential conflict of interest. The remaining authors declare that this study received funding from Thales Digital Solutions Inc. The funder was not involved in the study design, collection, analysis, interpretation of data, the writing of this article or the decision to submit it for publication.
